# Molecular identification and phylogenetic analysis of *Echinococcus vogeli* Rausch & Bernstein, 1972 from *Cuniculus paca* Linnaeus, 1766 consumed in extractive reserves in Acre, Brazil

**DOI:** 10.1007/s00436-026-08699-x

**Published:** 2026-05-22

**Authors:** F. Bittencourt-Oliveira, T. P. Dias-Correia, L. B. Neves, P. E. F. Teixeira, J. C. Moreira, T. C. Pereira, F. B. Almeida, R. Rodrigues-Silva, J. R. Machado-Silva

**Affiliations:** 1 Laboratório de Referência Nacional em Hidatidose, Laboratório de Parasitologia Integrativa e Paleoparasitologia, Instituto Oswaldo Cruz, Fundação Oswaldo Cruz, Rio de Janeiro, Rio de Janeiro Brasil; 2 Instituto Federal do Mato Grosso do Sul, Campo Grande, Mato Grosso do Sul Brasil; 3 Instituto Federal do Acre, Xapuri, Acre Brasil; 4 Laboratório de Helmintologia Romero Lascasas Porto, Departamento de Microbiologia, Imunologia e Parasitologia, Faculdade de Ciências Médicas (FCM), Universidade do Estado do Rio de Janeiro, Rio de Janeiro, Brasil

**Keywords:** *Echinococcus vogeli*, *Cuniculus paca*, PCR, Genetic diversity, Population genetics, Brazil

## Abstract

Hunting wild animals for consumption is a common practice in the Brazilian Amazon and contributes to the transmission of zoonotic diseases, including neotropical echinococcosis (NE), caused by *Echinococcus vogeli* and *Echinococcus oligarthra*. Despite the endemicity of *E. vogeli* in this region, molecular data on its genetic diversity remain limited. This study characterized *E. vogeli* isolates obtained from lowland pacas (*Cuniculus paca*) in extractive reserves located in Xapuri and Sena Madureira, Acre, Brazil, using mitochondrial (*cox1*) and nuclear (*p29*) markers. All 16 isolates were confirmed as *E. vogeli*. Analysis of *cox1* sequences revealed high similarity with reference sequences from the Brazilian Amazon, low nucleotide divergence (0–0.55%), and the presence of four haplotypes, including shared haplotypes between the two study areas. In contrast, no intraspecific variation was observed in the *p29* sequences. These findings indicate low genetic diversity and suggest a relatively homogeneous pattern among the analyzed isolates, which may reflect gene flow or retention of ancestral polymorphisms. This study expands the limited molecular dataset available for *E. vogeli*, including additional *p29* sequence data for this species, a molecular marker that remains poorly represented for *E. vogeli*, and provides relevant insights into its transmission dynamics in Amazonian environments.

## Introduction

The initial description of species within the genus *Echinococcus* Rudolphi, 1801 was based mainly on morphological characteristics and the parasite–host relationship. Subsequently, genetic and morphological differences among genotypes have been identified using techniques such as polymerase chain reaction (PCR), analysis of gene loci and sequencing of nuclear and mitochondrial DNA, along with epidemiological studies (Thompson 2017; Daipert-Garcia et al. 2019).

In Brazil, five species of the genus *Echinococcus* have been reported to infect humans: *Echinococcus oligarthra* and *Echinococcus vogeli*, both of which cause neotropical echinococcosis (NE) and are associated with the Amazon biome, with the latter also reported in the Cerrado biome (Neves et al. 2017; Bittencourt-Oliveira et al. 2018; Daipert-Garcia et al. 2019); *Echinococcus granulosus sensu stricto (s.s.)* (G1-G3); and *Echinococcus ortleppi* and *Echinococcus canadensis* (G7) (Neves et al. 2024), which are related to some regions of southern Brazil and are capable of causing cystic echinococcosis (CE) (Eckert and Thompson 2017).

NE is a parasitic infection reported in lowland pacas (*Cuniculus paca* Linnaeus, 1766) (D’Alessandro and Rausch 2008; Almeida et al. 2013; Bittencourt-Oliveira et al. 2018), when they ingest food or water contaminated with *E. vogeli* eggs, which are eliminated in the feces of the bush dog (*Speothos venaticus* Lund, 1842), the only natural definitive host of *E. vogeli* (Soares et al. 2014). Oncospheres cross the intestinal wall, enter the hepatic portal system, and lodge mainly in the liver, where they develop into cysts (Eckert and Thompson 2017). The liver and other possibly infected organs serve as sources of infection when hunters offer them food for domestic dogs. Once infected, dogs contribute to the contamination of peridomestic areas by eliminating helminth eggs in their feces (Neves et al. 2017), creating conditions conducive to human infection.

Molecular methods have been used to identify the species, genotypes, and haplotypes observed in *E. granulosus s.s.* and for the differential diagnosis of *Echinococcus granulosus sensu lato* and *Echinococcus multilocularis*, as well as to describe the emergence of two distinct populations of *E. oligarthra* from definitive hosts in Argentina (Arrabal et al. 2017).

Mitochondrial markers have been recognized as important in evaluating the structures of *Echinococcus* populations (Laurimäe et al. 2018). Additionally, nuclear markers, such as the gene encoding the *p29* antigen found in the hydatid cyst fluid of *E. granulosus s.l.*, are important, as they are possible candidates for producing vaccines to prevent echinococcosis (Siles-Lucas et al. 2017).

Genetic variability has already been proven and established for *E. granulosus s.l*. However, increased knowledge of possible variants in the genus *Echinococcus*, especially for the species *E. vogeli*, can directly contribute to control, prevention and diagnosis programs for NE.

Given the neglected status of NE and its persistence in human populations living in close contact with wildlife, investigating the molecular diversity of *E. vogeli* in natural hosts is essential. This study provided novel data from wild lowland pacas, contributing to the understanding of zoonotic transmission dynamics in the Brazilian Amazon. Therefore, we conducted a population genetic study of *E. vogeli* in pacas from the Brazilian Amazon rainforest, utilizing both mitochondrial and nuclear markers.

## Materials and methods

### Ethical aspects

This work was carried out with the approval of the Sistema de Informação e Autorização da Biodiversidade (SISBIO) under number 13373-1 of the permanent license issued on 11/19/2007 and under the authorization of the Instituto Chico Mendes de Conservação da Biodiversidade (ICMBio) under license number 68985-1.

### Sample collection and study areas

**Fig. 1:**
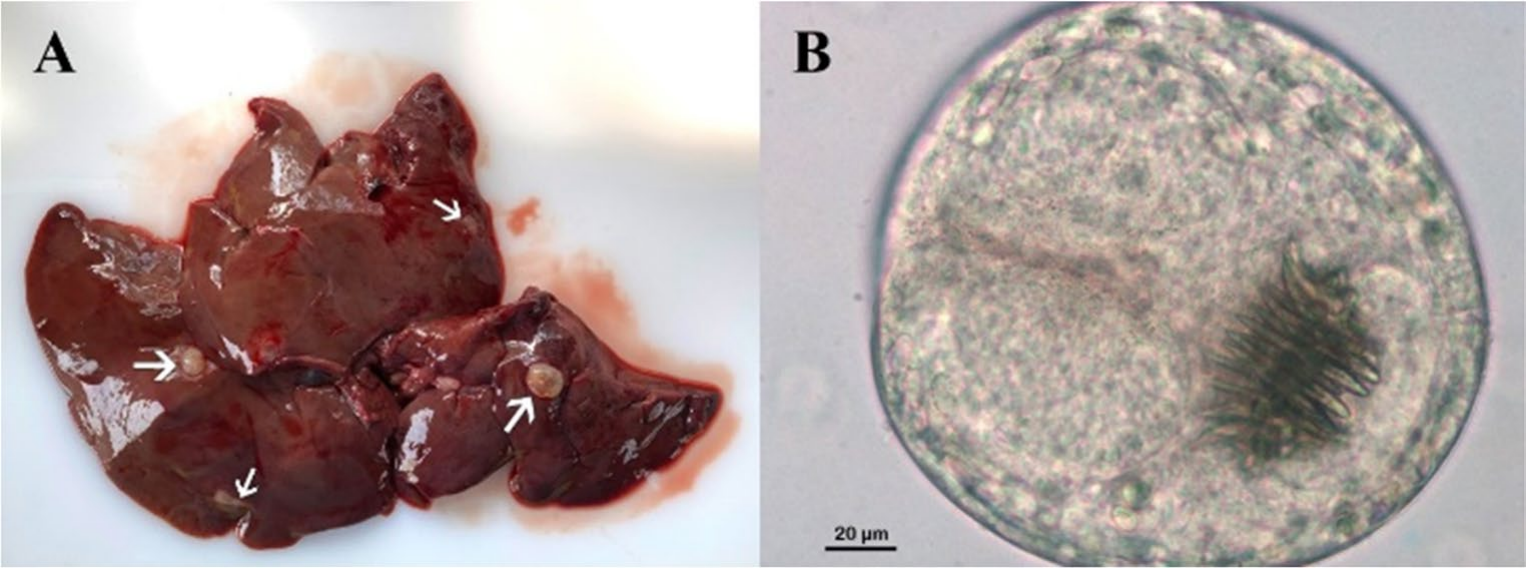
(**A**) Paca liver with characteristic cysts of *Echinococcus vogeli* infection; (**B**) *Echinococcus vogeli* protoscolices of hydatid fluid

The livers used in this study were donated by residents of agro-extractive forest reserves that practice subsistence paca hunting and are in the municipalities of Sena Madureira and Xapuri (Acre, Brazil). These livers were obtained between 2017 and 2019 to search for macroscopic lesions compatible with *Echinococcus vogeli* (Fig. 1).

Twenty-eight livers were examined, of which 16 presented cystic lesions compatible with *Echinococcus vogeli* infection. Multiple cysts were observed in infected livers; however, one cyst per liver was selected for molecular analysis.

### DNA extraction

Sixteen *E. vogeli* isolates were used for DNA extraction. The cysts were removed from the livers and stored in plastic tubes (15 mL) with 70% ethanol. Total DNA from each cyst was extracted via the QIAamp DNA Mini Kit (QIAGEN, Hilden, Germany) following the manufacturer’s instructions.

### Polymerase chain reaction (PCR): Cytochrome c oxidase subunit I (*cox1*) gene and *p29* antigen gene

A partial region (~ 450 bp) of the mitochondrial gene encoding subunit I of cytochrome c oxidase (*cox1*) was used for molecular analysis of *E. vogeli*, with forward 5’-TTTTTTGGGCATCCTGAGGTTTAT-3’ (CO1f) and reverse 5’- TAAAGAAAGAACATAATGAAAATG-3’ (CO1r) primers described by Bowles et al. (1992). A partial region (~ 222 bp) of the nuclear gene encoding the *p29* antigen was also used, with forward 5’-CATTGCATCTAAAGTCGTGG-3’ (*p29*f) and reverse 5’- ACATCAGAAGCTTCCGTCAG-3’ (*p29*r) primers (Santos et al. 2012). The PCR conditions for both genes were the same as those adopted by Santos et al. (2012). The PCR products were analyzed via electrophoresis on a 1% agarose gel in 1X TBE buffer (0.09 M Tris, 0.09 M borate, 0.02 M EDTA) at 80 V for 1.5 h. The gel was stained with GelRed, and the bands were visualized via a UV transilluminator.

### DNA sequencing

The amplicons were purified via the Illustra GFX PCR DNA and Gel Band Purification Kit (GE Healthcare) following the manufacturer’s instructions and then sequenced with the same PCR primers and the commercial ABI Prism BigDye Terminator Cycle Sequencing Kit (Applied Biosystems). The sequences were obtained via an ABI 3730 DNA Analyzer automatic sequencer from the RPT01A Automatic Sequencing subunit – IOC/RJ of the Fiocruz Technology Platforms Network. The obtained nucleotide sequences were aligned, edited, and assigned a consensus sequence via SeqMan (DNASTAR Inc.). The resulting sequences were compared (BLAST-NCBI) with *E. vogeli* sequences deposited in GenBank-NCBI.

### Phylogenetic analysis

Mitochondrial markers such as *cox1* have been widely used in phylogenetic studies of *Echinococcus* spp. (Thompson 2017; Eckert and Thompson 2017). Phylogenetic analyses of *E. vogeli* sequences were conducted after alignment, analysis of nucleotide composition, and calculation of the average intra- and interspecific distances between sequences via the ClustalW algorithm (Thompson 1994) in MEGA X v11.0.8 software (Tamura et al. 2021). Phylogenetic inference was performed using a Bayesian approach implemented in BEAST v1.10.4 after sequence alignment. The nucleotide substitution model was selected using jModelTest v2.1.10, which identified the Jukes–Cantor (JC) model as the best fit for the dataset. Markov chain Monte Carlo (MCMC) analyses were run for 10 million generations, with sampling every 1,000 generations, and the initial 10% of trees were discarded as burn-in using TreeAnnotator v1.10.4.

The dataset was restricted to representative sequences from medically relevant *Echinococcus* species. The following sequences available in GenBank were included: MK791154–MK791156, MK117958, MK117962–MK791164, MK791177, KX257618, KX527916, JX315616, and NC_009462 (*E. vogeli*); NC_009461 (*E. oligarthrus*); NC_044548 *(E. granulosus sensu stricto* G1); NC_011122 (*E. ortleppi*); NC_011121 (*E. canadensis*); and NC_000928 (*E. multilocularis*).

*Taenia solium* (NC_004022) was used as the outgroup to root the tree. The final tree was visualized and edited using the online platform iTOL v6. Only posterior probability values ≥ 0.95 were displayed at the nodes.

For the phylogenetic tree based on *p29*, the following GenBank sequences were used: KF528664 (*E. granulosus* s.s. G1); KF528665 (*E. equinus*), KF528666 (*E. ortleppi*); KF528667 (*E. canadensis* G6); KF528668 (*E. canadensis* G7); KF528669 (*E. canadensis* G10); and KF528673 (*E. multilocularis*).

### Molecular diversity

The molecular diversity indices of the number of segregating sites, the number and diversity of haplotypes, and the nucleotide diversity (π) were calculated with Dna SP software v. 5 (Librado and Rozas 2009), and Network software v.10.2 (Fluxus Technology Ltd 2021) was used to construct a median-joining flowchart of the relationships between haplotypes.

## Results

**Fig. 2:**
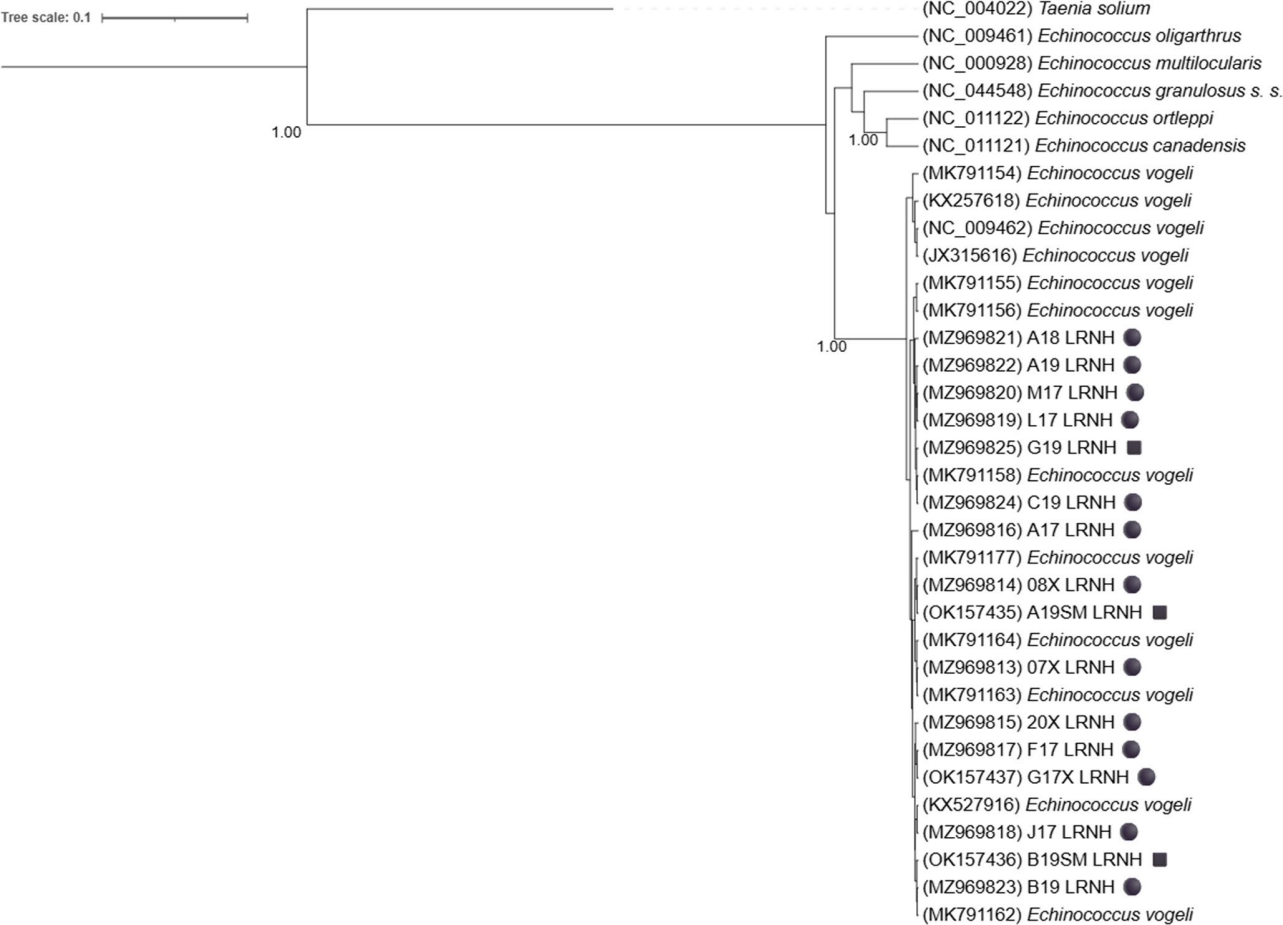
Phylogenetic tree inferred using a Bayesian approach implemented in BEAST, based on ~ 390 bp *cox1* sequences of *Echinococcus* samples obtained in the present study and reference sequences available in GenBank. *Taenia solium* was used as the outgroup. Posterior probability values ≥ 0.95 are shown at the nodes. The scale bar represents substitutions per site. Samples from Xapuri, Acre (accession numbers MZ969813–MZ969824; OK157437) are marked with circles, and those from Sena Madureira, Acre (accession numbers MZ969825; OK157435 and OK157436) are marked with squares

The sixteen *E. vogeli* isolates were positive by PCR for *cox1* and *p29*. The sequences obtained for *cox1* were compared with other sequences available in GenBank via the BLAST tool and demonstrated approximately 99% similarity and 100% coverage with other *E. vogeli* samples (KX527916; KX257618; NC_009462). The p29 sequences demonstrated similarities that ranged from 95% to 97% and showed 98% coverage with other species of the genus (KF528663 to KF528670; KF528672; KF528673), as no *p29* sequences of *Echinococcus vogeli* were available in GenBank at the time of analysis. The phylogenetic tree constructed from the *cox1* sequences corroborated the identification of the species *E. vogeli* (Fig. 2). All sequences generated in the present study formed a well-supported monophyletic clade together with other *E. vogeli* sequences available in GenBank, with maximum posterior probability support (1.00). No paraphyletic relationships were observed among the species included in the analysis.

**Fig. 3:**
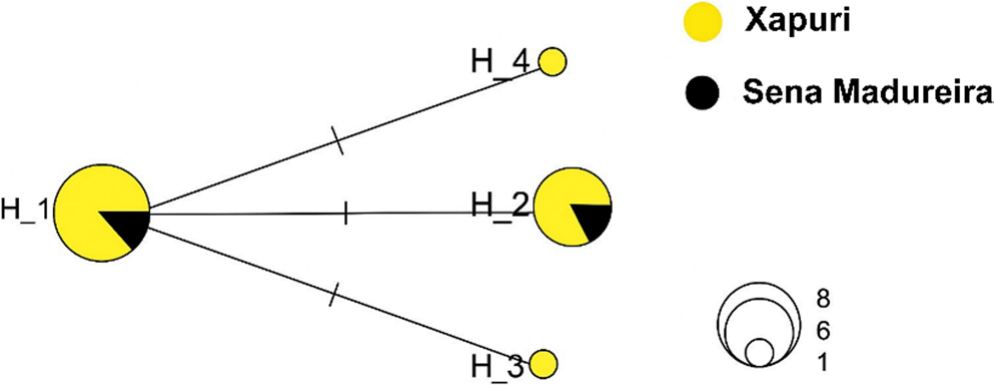
Parsimony haplotype network for *Echinococcus vogeli* based on analysis of *cox1* sequences (401 bp). ^a^ The frequency of each haplotype is indicated by the size of the circles, and mutational events are indicated by cross lines

The molecular divergence between the *E. vogeli* sequences ranged from zero to 0.55%. The analysis revealed three segregation sites containing four haplotypes, with H1 as the central haplotype, while H1 and H2 were shared between the two study sites (Fig. 3). Our total haplotype diversity (HD) and nucleotide diversity (π) indices were HD = 0.64167 and π = 0.00187, respectively (Table 1).

**Table 1:** Molecular diversity indices based on *cox1* for *Echinococcus vogeli* samples collected at two locations in the state of Acre, Brazil

Locality	*N*	S	NH	HD	π	Haplotype
Sena Madureira	3	1	2	0.66667	0.00166	H1, H2
Xapuri	13	3	4	0.67949	0.00204	H1, H2, H3, H4
*E. vogeli* (total)	16	3	4	0.64167	0.00187	
Sequences H1	OK157437, OK157436*, OK157435*, MZ969823, MZ969818, MZ969817, MZ969814, MZ969813					
Sequences H2	MZ969825*, MZ969824, MZ969822, MZ969821, MZ969820, MZ969819					
Sequences H3	MZ969816					
Sequences H4	MZ969815					

N: number of sequences; S: number of segregation sites; NH: number of haplotypes; HD: haplotype diversity; π: nucleotide diversity; *Sequences of samples from Sena Madureira

The partial sequences of the *p29* gene generated from all the samples in the present study did not present nucleotide differences.

## Discussion

Genetic data on *Echinococcus vogeli* remains scarce in the literature. This study added to only two previous Brazilian investigations (Santos et al. 2012; Daipert-Garcia et al. 2019) that have explored the population structure of this parasite. Our findings are consistent with both studies, particularly in demonstrating the low genetic variability of *E. vogeli* in Acre. The presence of shared haplotypes between geographically distinct populations may reflect the retention of ancestral polymorphisms and possible gene flow between geographically proximate areas. Although Santos et al. (2012) and Daipert-Garcia et al. (2019) relied on broader sampling and greater population-level divergence of *E. vogeli*, which encompasses distinct hosts and Amazonian regions, our samples presented genetic similarity with representative *E. vogeli* sequences from the Brazilian Amazon available in GenBank.

The phylogenetic analysis further supported the identification of the isolates as *E. vogeli*, with all sequences clustering in a well-supported monophyletic clade.

Although regions of the Brazilian Amazon rainforest are considered endemic for NE, there are few approaches that involve the collection and molecular characterization of neotropical *Echinococcus* species in their natural hosts (Santos et al. 2012; Almeida et al. 2013). Possible causes for such discrepancies include the lack of surveillance in areas where individuals are vulnerable to NE and the difficulty of studying parasites in wild animals (Santos et al. 2012).

Conceptually, when a parasite is maintained in a synanthropic cycle, the genotypes that infect humans are expected to be similar to those circulating in wild animals (Santos et al. 2012).This idea is supported by the low molecular divergence observed in *cox1* sequences of *E. vogeli* in our analyses, which corroborates the findings of Daipert-Garcia et al. (2019), who reported genetic variation ranging from 0 to 0.80% in *E. vogeli* populations from human cases, including those from our geographic area. In contrast, our study analyzed cysts obtained from wild pacas, which are natural intermediate hosts. By incorporating an additional host from the sylvatic environment, we expanded the molecular perspective of the synanthropic cycle of *E. vogeli*, where it is expected that the genotypes infecting humans do not differ from those circulating among wild animals. Similarly, our overall diversity indices were lower than those reported by Santos et al. (2012) (HD = N/A; π = 0.0044) and Daipert-Garcia et al. (2019) (HD = 0.796; π = 0.0037), reinforcing the notion of a genetically homogeneous population.

In this study, the *p29* gene served primarily as a conserved nuclear marker for species-level identification. To the best of our knowledge, this study provides the first publicly available *p29* sequence data for *Echinococcus vogeli* in GenBank (accession number PZ294616), helping to address a relevant molecular data gap for this species. Although no nucleotide variation was observed among the sequences, the availability of these data contributes to future comparative and evolutionary studies involving nuclear markers in *Echinococcus*. Although the genetic diversity of *E. vogeli* was low in our samples based on mitochondrial data, the identification of haplotypes from wild pacas provides additional support for a synanthropic transmission cycle involving species frequently consumed by rural communities. This finding reinforces the zoonotic risk and the need for molecular surveillance in Amazonian regions where human cases remain underreported. In this context, future studies integrating morphological and molecular data may provide a more comprehensive understanding of the intraspecific variation and population structure of *E. vogeli*.

## Conclusion

This study contributed to the limited knowledge on the population genetics of *Echinococcus vogeli* in the Brazilian Amazon by providing new molecular data based on mitochondrial markers. The *cox1* gene proved effective for characterizing haplotypes and revealed low genetic diversity among isolates from Acre. The molecular data generated here contribute to the understanding of *E. vogeli* circulation in natural hosts, supporting the need for targeted surveillance in regions where wildlife–human contact facilitates zoonotic transmission. Although the *p29* gene showed no intraspecific variability, which is consistent with its nuclear origin and the short fragments analyzed, this study provides additional *p29* sequence data for *E. vogeli*, contributing to the expansion of the molecular dataset for future comparative analyses. The sharing of haplotypes across populations is consistent with the possibility of ancestral polymorphism retention and gene flow within this region. Expanding the sampling effort, increasing the number of genetic markers, and exploring more variable nuclear regions will be essential in future studies to better understand the genetic structure and transmission dynamics of *E. vogeli* in neotropical environments.

## Data Availability

Nucleotide sequences obtained in this study are deposited in GenBank under accession numbers: MZ969813 to MZ969825, OK157435 to OK157437 and PZ294616.
